# Allelopathic Interactions Between the Green-Tide-Forming *Ulva prolifera* and the Golden-Tide-Forming *Sargassum horneri* Under Controlled Laboratory Conditions

**DOI:** 10.3390/plants13212966

**Published:** 2024-10-24

**Authors:** Ruibin Sun, Onjira Korboon, Wenfei Ma, Xingyue Ren, Xiaonan Wang, Narongrit Muangmai, Qikun Xing, Xu Gao, Jingyu Li

**Affiliations:** 1 Key Laboratory of Mariculture, Ministry of Education, Fisheries College, Ocean University of China, Qingdao 266003, China; sunruibin@stu.ouc.edu.cn (R.S.); onjira.ko@ku.th (O.K.); wisteria_ma@163.com (W.M.); renxingyue1998@163.com (X.R.); wxn2516@stu.ouc.edu.cn (X.W.); 2 Department of Fishery Biology, Faculty of Fisheries, Kasetsart University, Bangkok 10900, Thailand; narongrit.m@ku.ac.th; 3 Key Laboratory of Marine Genetics and Breeding, Ministry of Education, College of Marine Life Science, Ocean University of China, Qingdao 266003, China; qikunxing@ouc.edu.cn

**Keywords:** allelopathy, harmful algal blooms, *Sargassum horneri*, *Ulva prolifera*

## Abstract

Harmful algal blooms (HABs) represent a significant global marine ecological disaster. In the Yellow Sea, green and golden tides often occur simultaneously or sequentially, suggesting that interspecific competition involves not only spatial and resource competition but also allelopathy. This study investigated the allelopathic interactions between *Ulva prolifera* and *Sargassum horneri* using physiological and biochemical parameters, including relative growth rate (RGR), cell ultrastructure, chlorophyll fluorescence, enzyme activity, and metabolomics analysis. The results showed that *S. horneri* filtrate significantly inhibited *U. prolifera* growth, while *U. prolifera* filtrate had no significant effect on *S. horneri*. Both algal filtrates caused cellular damage and affected photosynthesis, enzyme activities, and metabolism. However, their allelopathic responses differed: *U. prolifera* may rely on internal compensatory mechanisms, while *S. horneri* may depend on defense strategies. These findings provide insights into the dynamics of green and golden tides and support the scientific control of HABs through allelopathy.

## 1. Introduction

Harmful algal blooms (HABs), caused by climate change, eutrophication, and the introduction of exogenous algae, significantly impact ecosystems and human health [1,2]. In recent years, the frequency of HABs in global freshwater and marine environments has increased [3,4]. The Yellow Sea and the East China Sea have been particularly prone to these events [5], with green tides (e.g., *Ulva prolifera*) and golden tides (e.g., *Sargassum horneri*) being the most common types [6]. Green tides predominantly occurred in the Yellow Sea, while golden tides are more frequently found in both the Yellow Sea and the East China Sea [7,8,9]. However, intermingling and coexistence of these algal types occurred during drifting [5]. From 2012 to 2014, green tides erupted in the Yellow Sea. During this period, significant occurrences of *S. horneri* were reported in both the Yellow and East China Seas, coexisting with floating *U. prolifera* [10]. Similarly, from April to June 2017, simultaneous occurrences of green, golden, and red tides were observed in the southern Yellow Sea at 35°N latitude [11]. The coexistence of green and golden tides leads to spatial and resource competition between them and potentially involves allelopathic interactions.

Allelopathy refers to the production and release of allelopathic substances by algae into the extracellular environment, which exert inhibitory or stimulatory effects on target species [12]. The effectiveness of allelopathy depends on the type and concentration of allelopathic substances produced, as well as the initial proportion of algal species, and exhibits species-specificity [13]. Under natural conditions, the effects of allelopathy may be influenced by competition and environmental factors, making the situation more complex [14]. Active substances in algal filtrates (referring to the culture medium containing substances released by algae during growth), rather than algal cells, are considered more suitable for reflecting allelopathic effects [15]. For example, Mao et al. [16] observed that the filtrate from *Alexandrium pacificum* inhibited the growth of *Thalassiosira pseudonana*, confirming allelopathy between these species. Guo et al. [17] demonstrated the allelopathic effects of *Skeletonema costatum* on *Karenia mikimotoi* by adding algal filtrates and extracts. Xu et al. [12] found that cell-free filtrates of *Cylindrotheca closterium* contained substances that inhibited or lysed *Prorocentrum donghaiense*, further confirming allelopathy.

Allelopathy inhibits algal growth and development by affecting photosynthesis, damaging cell structures, altering enzyme activity, and disrupting other physiological processes [18]. This mechanism is crucial for algae to gain competitive advantages, maintain clear water conditions, and sustain ecological balance [19]. *Aureococcus anophagefferens* and *Aureoumbra lagunensis* have been shown to inhibit phytoplankton growth by targeting the photosystems of these organisms, highlighting the potential role of allelopathy in influencing coastal phytoplankton community dynamics [20]. Mohamed and Al Shehri [21] demonstrated that the allelopathic effects of *Stratiotes aloides* significantly inhibit the growth rate, alkaline phosphatase activity, and toxicity of planktonic cyanobacteria. Fistarol et al. [22] found that toxins produced by the allelopathic effects of *Prymnesium parvum* could significantly impact co-occurring plankton species, altering community structures.

Since 2008, simultaneous or successive outbreaks of green and golden tides have been observed in the same marine areas. Based on the patterns of community succession, researchers hypothesize that, in addition to spatial and resource competition, allelopathy may also mediate the competitive relationships between these algae. Although allelopathy is recognized as an important factor in aquatic ecosystems, few studies have investigated the interspecific allelopathic interactions between green and golden tides. This study aims to investigate whether allelopathy exists between *U. prolifera* and *S. horneri*, elucidate the mechanisms of these interactions, aiming to provide theoretical foundations for understanding the succession and dynamics of green and golden tide communities, and offer new insights for the biological control of HABs.

## 2. Results

### 2.1. Relative Growth Rate (RGR)

**Figure 1:**
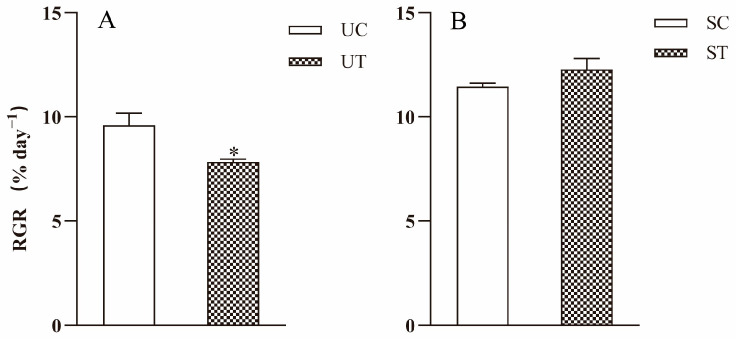
RGR of *U. prolifera* and *S. horneri* cultured in different media for 9 days. Data are presented as mean ± SE (*n* = 3). The asterisks indicate significance between the treatment group and the control group (*p* < 0.05). (**A**) Relative growth rates of *U. prolifera* in the control (UC) and treatment (UT) groups. (**B**) Relative growth rates of *S. horneri* in the control (SC) and treatment (ST) groups.

The RGR of *U. prolifera* was significantly different between the UT and UC groups (*p* = 0.008) (Figure 1A). The RGR of UT group was 7.84% day^−1^, which was significantly lower compared to that of the UC group (9.59% day^−1^). The RGR of *S. horneri* showed no significant difference between the ST and SC groups (*p* = 0.062) (Figure 1B). The RGR of ST group was 12.28% day^−1^, marginally higher than that of SC group (11.45% day^−1^).

### 2.2. Cell Ultrastructure

**Figure 2:**
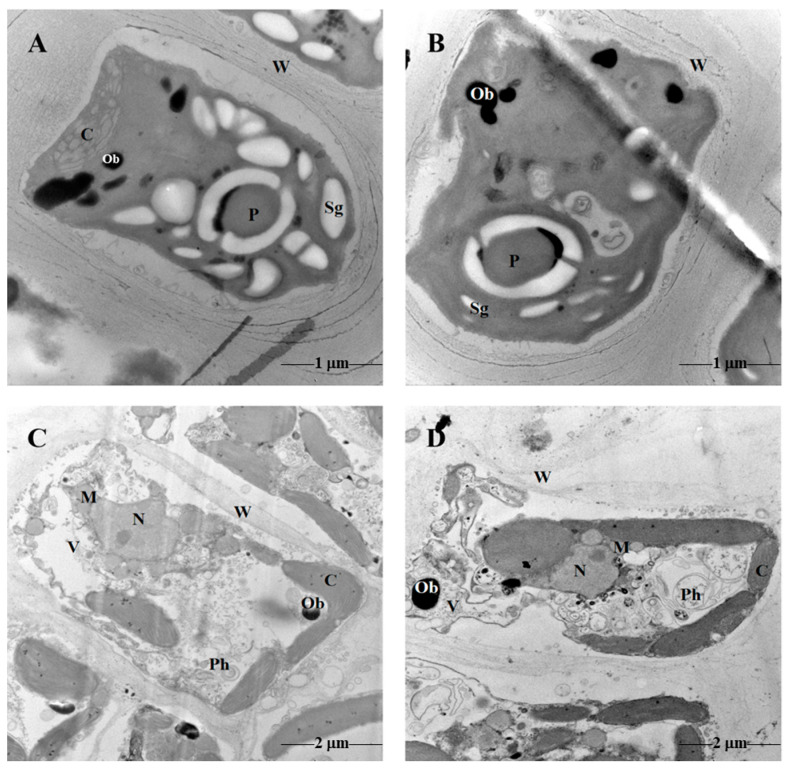
Ultrastructures of *U. prolifera* and *S. horneri* cells cultured in different media for 9 days. Details of *U. prolifera* ultrastructure in UC (**A**) and UT (**B**) groups. Details of *S. horneri* ultrastructure in SC (**C**) and ST (**D**) groups. W: cell wall; C: chloroplast; P: pyrenoid; Sg: starch granule; Ob: osmiophilic body; N: cell nucleus; M: mitochondria; V: vacuole; Ph: physode.

Ultrastructures of *U. prolifera* and *S. horneri* cells cultured for 9 days with algae filtrate are shown in Figure 2. Compared with UC group, *U. prolifera* in UT group exhibited disrupted intracellular membrane structures, with structures similar to autophagosomes observed. Additionally, chloroplasts in UT group were not prominently visible within the cell, and there was a noticeable reduction in starch granules. However, the variation in the number of osmiophilic bodies was not obvious (Figure 2A,B).

Notable alterations in cellular morphology were observed in ST group compared to SC group. The vacuoles became smaller, the number of chloroplasts remained relatively stable, but the thylakoids appeared blurred. Additionally, the number and size of physodes increased, while the number of osmiophilic bodies also increased (Figure 2C,D).

### 2.3. Chlorophyll Fluorescence

**Figure 3:**
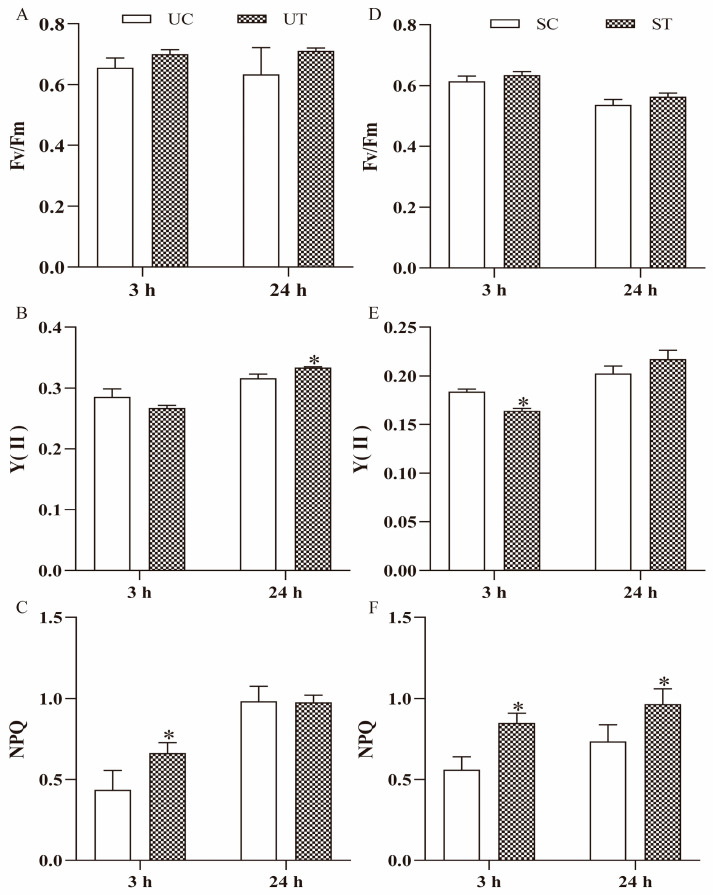
Chlorophyll fluorescence parameter of *U. prolifera* and *S. horneri* cultured in different medium for 3 h and 24 h. Data are presented as mean ± SE (*n* = 3). The asterisks indicate significance between the treatment group and the control group (*p* < 0.05). (**A**) Fv/Fm of *U. prolifera* in the UC and UT groups. (**B**) Y(II) of *U. prolifera* in the UC and UT groups. (**C**) NPQ of *U. prolifera* in the UC and UT groups. (**D**) Fv/Fm of *S. horneri* in the SC and ST groups. (**E**) Y(II) of *S. horneri* in the SC and ST groups. (**F**) NPQ of *S. horneri* in the SC and ST groups.

The maximum quantum efficiency of photosystem II (Fv/Fm) was slightly higher in UT group than UC group at 3 h and 24 h, but the difference was not statistically significant (*p* > 0.05) (Figure 3A). No significant difference was observed in the effective quantum yield of photosystem II (Y(II)) between the UT and UC groups at 3 h (*p* = 0.079). However, after 24 h, Y(II) in UT group was significantly higher than that of the UC group (*p* = 0.012) (Figure 3B). Non-photochemical quenching (NPQ) was significantly higher in the UT group than the UC group after 3 h (*p* = 0.044), but no significant difference was observed after 24 h (*p* = 0.924) (Figure 3C).

Fv/Fm was higher in the ST group than in the SC group at 3 h and 24 h, though the difference was not significant (*p* > 0.05) (Figure 3D). Y(II) in the ST group was significantly lower than that in the SC group at 3 h (*p* = 0.0007) (Figure 3E), but no significant difference was observed at 24 h (*p* = 0.098) (Figure 3E). NPQ was significantly higher in the ST group than the SC group at 3 h and 24 h (*p* < 0.05) (Figure 3F).

### 2.4. Enzyme Activities

**Figure 4:**
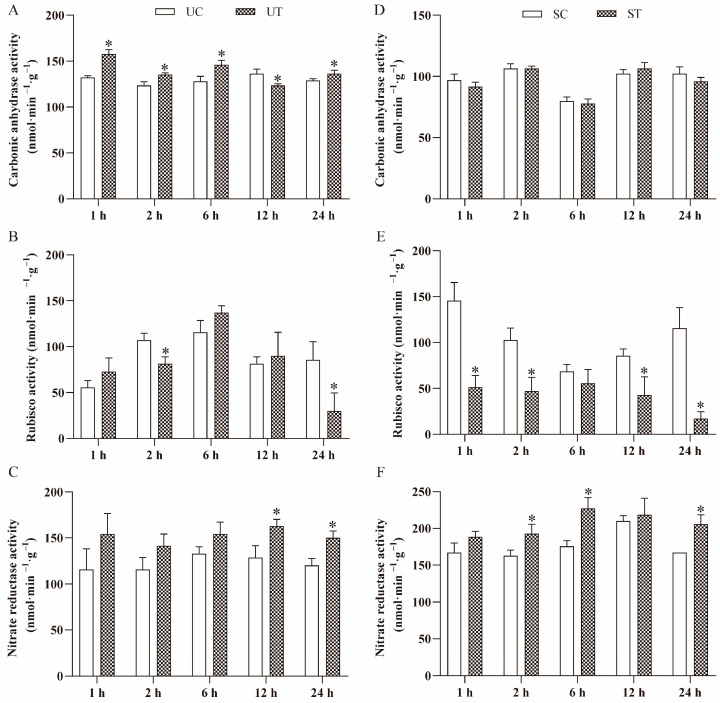
Changes in activities of carbonic anhydrase (CA), rubisco and nitrate reductase (NR) of *U. prolifera* and *S. horneri* cultured in different medium for 1 h, 2 h, 6 h, 12 h and 24 h. Data are presented as mean ± SE (*n* = 3). The asterisks indicate significance between the treatment group and the control group (*p* < 0.05). (**A**) CA activity of *U. prolifera* in the UC and UT groups. (**B**) Rubisco activity of *U. prolifera* in the UC and UT groups. (**C**) NR activity of *U. prolifera* in the UC and UT groups. (**D**) CA activity of *S. horneri* in the SC and ST groups. (**E**) Rubisco activity of *S. horneri* in the SC and ST groups. (**F**) NR activity of *S. horneri* in the SC and ST groups.

*S. horneri* filtrate significantly influenced CA activity in *U. prolifera*. Except for 12 h, CA activity in the UT group was consistently higher than that in the UC group (*p* < 0.05) (Figure 4A). In contrast, CA activity between the ST and SC groups showed almost no difference (Figure 4D). Over the course of the experiment, both the SC and ST groups exhibited fluctuating CA activity patterns, with the lowest values occurring at 6 h. Additionally, overall CA activity in *S. horneri* was lower compared to *U. prolifera*.

Rubisco activity in UT group was significantly lower than that in the UC group after 2 h and 24 h (*p* < 0.05), with no significant differences noted at other time points (Figure 4B). Excluding 6 h, Rubisco activity in ST group was significantly lower than in the SC group (*p* < 0.05) (Figure 4E). As the culture time increased, Rubisco activity in both the UC and UT groups demonstrated an initial increase followed by a decrease, whereas in the SC group, the activity initially decreased before increasing.

NR activity in the UT group was generally higher than in the UC group, with particularly significant differences after 12 h and 24 h (*p* < 0.05) (Figure 4C). NR activity in ST group was consistently higher than in SC group throughout the experiment (*p* < 0.05). Both ST and SC groups exhibited a slight increasing trend in NR activity over time (Figure 4F). Additionally, NR activity in *S. horneri* was significantly higher than in *U. prolifera* at these observed time points.

### 2.5. Algal Metabolomics

To elucidate the response mechanisms of *U. prolifera* and *S. horneri* to allelopathy, samples collected after 9 days of culture in algal filtrate were used for metabolomic analysis. The quality control (QC) samples from both experiments exhibited high aggregation, indicating good stability of instrument detection and experimental reproducibility, ensuring the high-quality data for subsequent analysis. The general overview of the samples was obtained through the principal component analysis (PCA) and partial least squares discriminant analysis (PLS-DA) of the QC samples. As shown in Figure S1, PCA displayed clear distributions among the groups, and PLS-DA showed consistent results. Moreover, the R2 and Q2 values were close to 1, indicating the reliability of the model to reflect the true condition of the samples. Differential metabolites (DMs) of *U. prolifera* and *S. horneri* were screened based on VIP > 1 (OPLS-DA) and *p* < 0.05 (Student’s *t*-test) (Tables S1 and S2). Additionally, volcano plots were generated to visually display the overall distribution of DMs (Figure S2).

**Figure 5:**
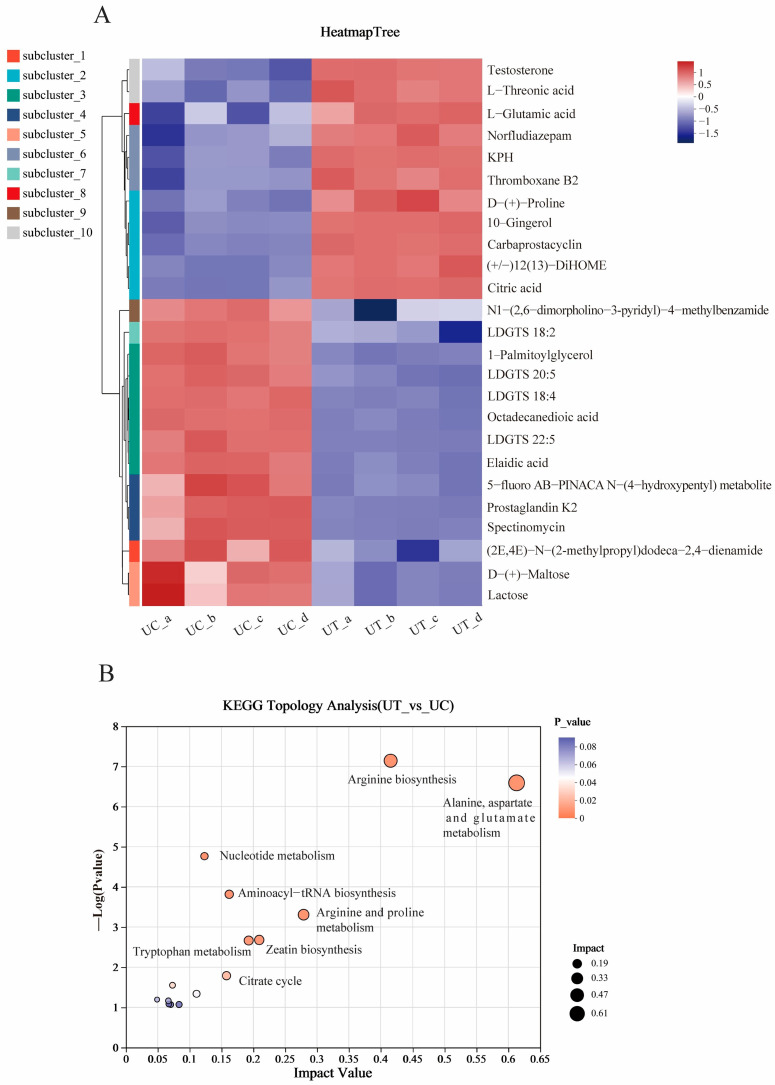
Clustering heat map of metabolites in *U. prolifera* (**A**); KEGG pathway analysis of significantly changed metabolites in *U. prolifera* (**B**).

Compared to the UC group, the clustered heatmap of the top 25 DMs in the UT group showed significant changes (11 metabolites increased, 14 metabolites decreased) (Figure 5A). In the UT group, the content of amino acid metabolites such as L-Threonic acid, L-Glutamic acid, L-Aspartic acid, and DL-Arginine were significantly accumulated while the content of 1-Palmitoylglycerol and Octadecanedioic acid were decreased. KEGG topological analysis also revealed that the disturbed metabolic pathways were mainly involved in alanine, aspartate, and glutamate metabolism and arginine biosynthesis, as well as arginine and proline metabolism (Figure 5B).

**Figure 6:**
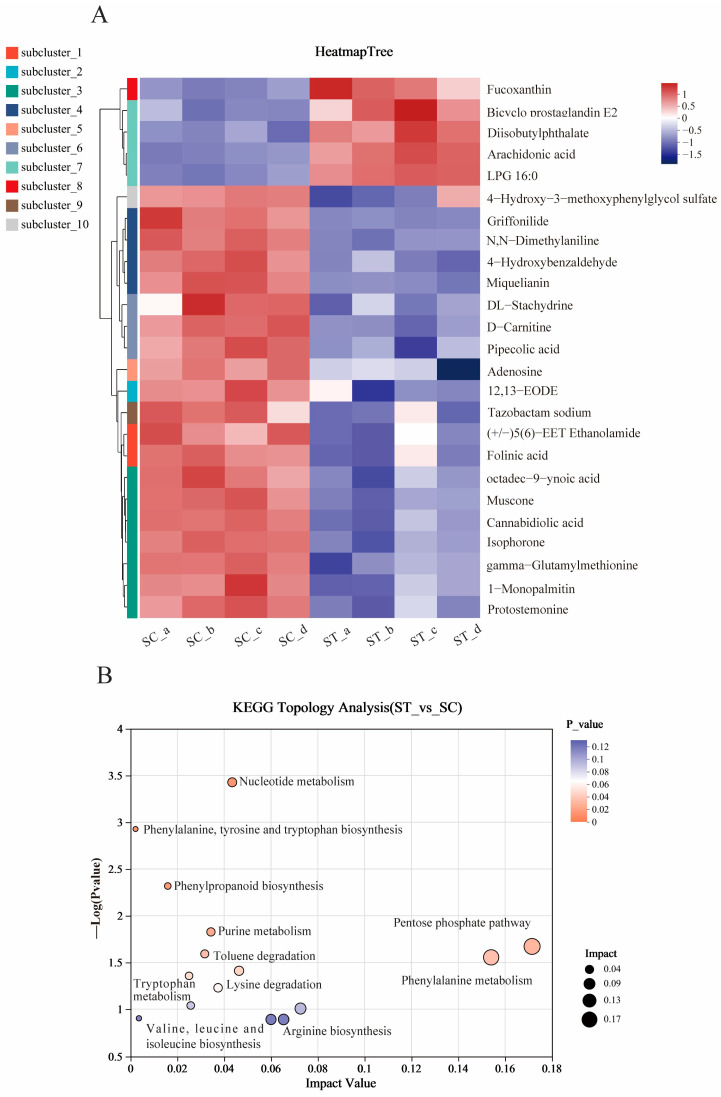
Clustering heat map of metabolites in *S. horneri* (**A**); KEGG pathway analysis of significantly changed metabolites in *S. horneri* (**B**).

In contrast to the metabolites of *U. prolifera*, although the top 25 DMs in the ST group also showed significant changes compared to the SC group, the clustered heatmap indicated that most DMs in the ST group were downregulated (5 metabolites upregulated, 20 downregulated) (Figure 6A). The content of Arachidonic acid, Fucoxanthin, and Uridine 5′-monophosphate (UMP) were significantly accumulated in the ST group while the content of Folinic acid, 1-Monopalmitin, and L-Phenylalanine were decreased. KEGG pathway analysis further indicated that the affected metabolic pathways were mainly associated with phenylalanine, tyrosine, and tryptophan biosynthesis, nucleotide metabolism and the pentose phosphate pathway (Figure 6B).

## 3. Discussion

The investigation of allelopathy is crucial for a deeper understanding of HABs. The succession of dominant phytoplankton species plays a significant role in the occurrence of HABs, which are dynamically influenced by allelopathy [23]. Certain species of bloom-forming algae can release allelopathic substances that inhibit the growth of other algae under the same resource conditions [24]. Current research on allelopathy has primarily focused on interactions between microalgae, and between macroalgae and microalgae, with few reports on allelopathy between two bloom-forming macroalgae. Therefore, this study combined metabolomics with conventional physiological and biochemical measurements to explore the allelopathic effects and mechanisms between *U. prolifera* and *S. horneri* for the first time. These findings provide a theoretical basis for understanding the dynamics and succession patterns of green and golden tides, and offer insights for the biological control of HABs.

### 3.1. S. horneri Filtrate Caused Photosystem Disruption in U. prolifera

The results of our study suggested that *S. horneri* filtrate had negative impacts on the photosystem of *U. prolifera*. Ultrastructure analysis revealed that treatment with *S. horneri* filtrate damaged the cellular membrane structures of *U. prolifera*, particularly affecting the chloroplasts and reducing starch granule numbers within cells. These changes are likely due to chloroplast damage, resulting in decreased photosynthesis rates and starch accumulation [25], or the rapid transport and utilization of photosynthetic assimilates in other tissues [26]. Changes in enzyme activities and metabolomics further supported these observations.

In this study, the *S. horneri* filtrate notably inhibited photosynthesis-related enzyme activities in *U. prolifera*, particularly Rubisco, which is crucial for the carbon fixation in photosynthesis [27]. Under stress, the activity of Calvin cycle-related enzymes, like Rubisco, may decrease due to energy reallocation and physiological adjustments [28]. Oxidative stress may have further impaired the photosynthetic mechanisms of cells, and photosynthesis directly affected carbohydrate production [29]. Therefore, to cope with this stress, *U. prolifera* likely hydrolyzed starch into monosaccharides through enzymatic reactions, and utilized the pentose phosphate pathway to produce NADPH. NADPH supplied the necessary electrons for the cyclic electron transport chain of Photosystem I (PSI), thereby maintaining photosynthetic activity under adverse conditions [28]. Metabolomic analysis revealed that DMs in the pentose phosphate pathway were significantly upregulated, further confirming the capability of *U. prolifera* to adjust its energy metabolism pathways in response to stress. This adjustment also explained the observed reduction in starch granules in ultrastructure of the *U. prolifera* treated group.

Additionally, changes in chlorophyll fluorescence parameters, enzyme activities, and metabolomics of *U. prolifera* indicated the impact of *S. horneri* filtrate on the photosynthetic system of *U. prolifera* and revealed the chemical defense mechanisms exhibited by *U. prolifera* under allelopathic effects. Under the influence of *S. horneri* filtrate, Fv/Fm, Y(II), and NPQ in *U. prolifera* exhibited varying enhancements. This was especially notable in NPQ during the initial culture stages and in Y(II) later on. These enhancements were likely due to the exposure to allelopathic chemicals from *S. horneri* induced stress, which triggered an increase in photoprotective mechanisms to protect the photosynthetic system from damage [30].

CA participates in the carbon concentrating mechanism, increasing the concentration of CO_2_ around Rubisco, thereby enhancing carbon fixation efficiency [31]. Macroalgae commonly utilize bicarbonate through the catalytic action of carbonic anhydrase, a prevalent method of inorganic carbon utilization. *U. prolifera* exhibited strong inorganic carbon utilization capabilities, possessing robust intracellular and extracellular CA activities [32]. The results showed that the filtrate from *S. horneri* significantly enhanced CA activity in *U. prolifera*. This might be due to June being the outbreak period for *U. prolifera*. There was competition for carbon sources among *U. prolifera* individuals and with other floating seaweeds such as *S. horneri*. *S. horneri* filtrate promoted CA activity in *U. prolifera*, suggesting that allelochemicals from *S. horneri* might further stimulate the competitive potential for carbon sources in *U. prolifera*. Concurrently, *U. prolifera* might have enhanced its inorganic carbon concentrating mechanism to counteract the adverse effects of the allelochemicals from *S. horneri*.

In addition to carbon metabolism, our results also indicated that nitrogen metabolism also participate in the response of *U. prolifera* to allelopathy induced by *S. horneri* filtrate. NR acts as the initiating and rate-limiting enzyme in the nitrogen assimilation process [33]. Under environmental stress, NR activity in the *U. prolifera* treatment group was significantly increased to enhance its nitrogen uptake and assimilation capabilities, thereby maintaining optimal metabolic processes [34]. Amino acids are not only the building blocks of proteins and polypeptides but also serve as precursors for various metabolites, playing multiple roles in algae growth and other biological processes [35]. In this study, five DMs in the amino acid metabolic pathways of *U. prolifera* were upregulated under the treatment of *S. horneri* filtrate. This indicated an acceleration amino acid synthesis or protein degradation, suggesting that *U. prolifera* had adjusted its metabolic pathways to enhance antioxidant capacity and maintain normal physiological and metabolic functions in response to environmental stress [36] Amino acids were also part of the aminoacyl-tRNA biosynthesis pathway. The increased abundance of amino acids suggested that the *S. horneri* filtrate affected the growth and metabolism of *U. prolifera* [35]. UDP-glucose, a substrate for cellulose synthesis [37], was upregulated in the *U. prolifera* treated group, possibly indicating that under allelopathy, *U. prolifera* cells prioritized resource allocation to defense mechanisms, such as cell wall synthesis. Additionally, the downregulation of pheophorbide A in the chlorophyll degradation pathway might reflect a compensatory effect of *U. prolifera* against the allelopathy induced by *S. horneri* filtrate [38]. The downregulation of tryptophan, a precursor of auxins, likely led to inhibited growth [39]. Ultimately, the allelopathy induced by *S. horneri* filtrate inhibited the growth of *U. prolifera*, which was consistent with the findings of Cai et al. [40].

### 3.2. S. horneri Had a Specific Defense Response to the Allelopathic Effect of U. prolifera Filtrate

Ultrastructure analysis showed that *U. prolifera* filtrate also caused damage to the cellular membrane structures of *S. horneri*, particularly to the thylakoids, directly affecting photosynthetic efficiency [18]. Thylakoids, as key structures in photosynthesis [41], may directly lead to decreased photosynthetic ability if their structural integrity is compromised. Enzyme activity analysis indicated that Rubisco activity was significantly inhibited, suggesting that allelochemicals may have directly affected the photosynthetic physiological functions of *S. horneri*. Additionally, metabolomics data showed significant downregulation of phenylalanine and methyl jasmonate (MeJA), further revealing the complex effects of allelopathy on the photosynthetic system. Phenylalanine serves as a precursor for carotenoid biosynthesis. Carotenoids bind to associated with PSI and Photosystem II (PSII), thereby contributing to protection against photooxidative damage [42]. MeJA regulated the expression of genes associated with PSII, aiding cells in coping with photooxidative stress [43]. Therefore, the downregulation of phenylalanine and MeJA may weaken the photoprotective mechanisms within *S. horneri* cells, further suggesting that the blurred layering of thylakoids could impair the photosynthetic system.

Although *U. prolifera* filtrate had a significant negative impact on ultrastructure and photosynthetic system of *S. horneri*, the growth of *S. horneri* was not significantly affected as it was in *U. prolifera*. This may be attributed to the unique defensive mechanisms of *S. horneri*. Vacuoles play a regulatory role in the cellular environment [44]. The vacuoles in *S. horneri* cells treated with *U. prolifera* filtrate decreased in size, likely to regulate cellular water and ion balance [45]. Physodes primarily store polyphenolic compounds, associated with defense and antioxidation [46]. The increase in both the number and size of physodes in *S. horneri*-treated cells suggested an essential mechanism for enhancing antioxidative defense and adapting to environmental stress to sustain growth. Additionally, *U. prolifera* is composed of a single cell layer [47], while *S. horneri* exhibits a more complex multicellular structure that includes an epidermis, cortex, and medulla cells, along with plasmodesmata that facilitate intercellular material exchange [48]. This architecture may provide *S. horneri* with enhanced protection and help maintain its physiological functions. Therefore, when facing environmental challenges such as allelopathy, *S. horneri* may exhibit stronger survival and adaptability. This hypothesis merits further investigation to more comprehensively understand how different structural configurations influence algal survival strategies.

Changes in chlorophyll fluorescence parameters, enzyme activities, and metabolomics of *S. horneri* further demonstrated its chemical defense and adaptability. *U. prolifera* filtrate suppressed Y(II) in *S. horneri* but enhanced NPQ. This suggested that *S. horneri* may have experienced limitations in its actual capture efficiency of primary light and electron transfer capacity within a short timeframe. However, *S. horneri* may have responded to stress by increasing thermal dissipation to adapt to changes in the external environment [44]. Photosynthesis is coupled with the nitrogen assimilation process, likely due to the competition between the two processes for ATP, NADPH, and intermediate metabolites [49]. This adjustment might make cells more inclined to use limited energy and reducing power to support essential survival mechanisms, explaining why NR activity increased relative to Rubisco activity in the treated groups. Additionally, NR activity in *S. horneri* was generally higher than in *U. prolifera*, indicating its efficiency in the nitrogen assimilation process, which may enable it to exhibit stronger resistance to environmental stress [50]. These factors, along with the multicellular structure of *S. horneri*, may explain why its growth was not inhibited under allelopathic conditions.

Metabolomic results indicated that the upregulation of arachidonic acid in *S. horneri* under treatment with *U. prolifera* filtrate may be due to membrane damage caused by allelopathy. This response suggested a cellular strategy to maintain membrane integrity and fluidity, potentially through increased arachidonic acid synthesis for repair [51]. Pyrimidine nucleotides are crucial components of nucleic acids [52]. UMP plays a pivotal role at key points in pyrimidine nucleotide biosynthesis and nucleotide metabolism pathways [53]. Elevated UMP levels in *S. horneri* under allelopathy likely enhanced nucleic acid synthesis and repair capabilities to adapt to environmental pressures. Fucoxanthin exhibits high antioxidant activity by reducing free radicals [54]. Under allelopathic conditions, *S. horneri* might enhance fucoxanthin synthesis to protect its cells from oxidative stress damage.

### 3.3. Implications of Allelopathy for Future HABs Dynamics

The allelopathy between *U. prolifera* and *S. horneri* showed complexity on multiple biological levels including growth, photosynthesis, cell structure, enzyme activity, and metabolites. The results indicated that *S. horneri* filtrate significantly inhibited the growth of *U. prolifera*, whereas *U. prolifera* filtrate had no significant effect on the growth of *S. horneri*. Both types of algal filtrate significantly affected the cellular structure, photosynthetic parameters, and cellular metabolism of the other algal, although their mechanisms of response to allelopathy differed. *U. prolifera* appeared to cope with allelopathic stress mainly through internal compensatory mechanisms, while *S. horneri* primarily relied on its defense strategies. These findings highlighted the different adaptive strategies of the two algae under allelopathic conditions and underscored the competitive advantage of *S. horneri* in these ecological dynamics.

Since 2012, the biomass of *S. horneri* in the Yellow Sea and the East China Sea has been increasing. This expansion not only led to intermingling with *U. prolifera* but also resulted in a significant increase in several sea areas such as Qingdao and Rizhao, intensifying competition with native algae [10]. Additionally, Qi et al. [55] showed that the biomass of *S. horneri* continued to expand annually and was predicted to further increase in the future. Additionally, previous research by our team indicated that co-culture of *S. horneri* with *Gracilariopsis lemaneiformis* significantly promoted the growth of *S. horneri* [56]. In acidified and eutrophic ocean, *S. horneri* demonstrated a clear competitive advantage over *Saccharina japonica* [57]. In competition with cultivated *Undaria pinnatifida*, *S. horneri* also showed a more pronounced growth advantage [58]. These findings highlighted the strong adaptability and competitiveness of *S. horneri* across various environmental conditions. Consequently, the golden tides caused by *S. horneri* had become a significant ecological threat to coastal environments and aquaculture.

These findings underscore the complex dynamics of allelopathy between *U. prolifera* and *S. horneri*, highlighting the adaptability and competitiveness of *S. horneri* in various environmental conditions. Given the increasing biomass of *S. horneri* and its potential impact on coastal ecosystems, we predict that the golden tide induced by *S. horneri* may exert significant effects on HABs in the Yellow Sea and East China Sea in the future. Our results deepen the understanding of how allelopathy influences the interactions between these two species and provide a theoretical foundation for developing effective management strategies.

## 4. Materials and Methods

### 4.1. Algae Collection and Maintainence

The collection of *U. prolifera* and *S. horneri* was conducted during the green tide outbreak. *U. prolifera* samples were collected in June 2022 from the waters near Xiaomai Island (120°26′ E, 36°03′ N) and Lingshan Island (120°11′ E, 35°46′ N) in Qingdao, China, with a water temperature ranging from 19 to 20 °C. Observations under an optical microscope indicated that the predominant cells of *U. prolifera* were in the reproductive phase, characterized by germ cell cytocysts filled with reproductive cells [59] (Figure S3A).

The floating-type *S. horneri* was collected in June 2022 from the kelp aquaculture area of Shandong Rongcheng Li Island Marine Technology Co., Ltd. (122°35′ E, 37°15′ N). The water temperature was recorded as 19 °C. External morphological examinations revealed that *S. horneri* in June were in the reproductive phase with receptacles (Figure S3B).

Samples of *U. prolifera* and *S. horneri* were expeditiously transported to the laboratory in insulated plastic foam containers to maintain low-temperature conditions. Algal specimens demonstrating optimal growth were selected, and then were meticulously cleansed with sterilized filtered seawater to remove any attached substances. The algae were temporarily cultured in transparent plastic containers for one day at 19 °C, under a light intensity of 100 µmol m^−2^ s^−1^ with a 12/12 h light/dark cycle using LED white light. During this period, the algae were kept in sterilized filtered seawater. After the temporary culture, they were used for subsequent experiments.

### 4.2. Preparation of Algal Filtrates

The filtrates from *U. prolifera* and *S. horneri* were prepared based on the statistical data of algal biomass per unit volume of water during the outbreaks of green tide and golden tide, respectively [11]. Specifically, *U. prolifera* density was maintained at 40 g wet weight L^−1^, while *S. horneri* density was set at 30 g wet weight L^−1^. Under controlled conditions as described above, fresh intact algae were cultured with aeration in sterilized filtered seawater at the specified densities for 24 h. After culture, the solution was filtered through 0.45 µm membranes, and the filtrate was used immediately for subsequent experiments.

### 4.3. Culture Experiment

An experiment was conducted over 9 days using 8 L glass tanks, each containing 6 L of either prepared algal filtrate or sterilized filtered seawater. A total of 30 g of well-grown *U. prolifera* was placed in tanks with *S. horneri* filtrate as treatment group (UT group), while the corresponding control group used the same amount of *U. prolifera* placed in sterilized seawater (UC group). Similarly, 30 g of well-grown *S. horneri* was placed in tanks with *U. prolifera* filtrate as treatment group (ST group). The same amount of *S. horneri* was placed in sterilized seawater as control group (SC group). To standardize nutrient levels across all groups, sodium nitrate (AR, Tianjin Bodi Chemical Co., Ltd., Tianjin, China) and potassium dihydrogen phosphate (AR, Tianjin Hengxing Chemical Reagent Co., Ltd., Tianjin, China) were added into seawater to achieve final concentrations of 10 mg L^−1^ nitrogen and 1 mg L^−1^ phosphorus. Each experimental condition had three replicates.

To monitor the changes in photosynthesis, chlorophyll fluorescence parameters were measured using algae samples randomly collected from each group at 3 h and 24 h. For measurements of enzyme activities related to carbon and nitrogen assimilation in algae, samples were randomly collected from each condition at 2 h and 24 h. At the end of culture, algae samples were collected for the measurement of RGR and observation of algal cells ultrastructure. The remaining samples were preserved at −80 °C for the subsequent metabolomic analysis.

### 4.4. Measurement of RGR

The RGR of the algae was calculated using the following formula:

RGR (% d^−1^) = (ln W_t_ − lnW_0_)/t × 100% (1)

where W_t_ and W_0_ were the wet weights of algae at the end and beginning of the experiment, respectively, while t was the duration of culture experiment.

### 4.5. Observation of Ultrastructure

Upon completion of the culture experiment, a subset of algae samples from both the control and treatment groups was randomly selected. Tissue blocks, each less than 1 cubic centimeter, were carefully excised using razor blades and promptly transferred to a 0.1 mol L^−1^ glutaraldehyde fixative solution which was prepared in phosphate buffer (pH 7.2) for light-avoidance and low-temperature preservation. Samples were rinsed three times with 0.1 M phosphate buffer, followed by fixation in a 1% (*v*/*v*) osmium tetroxide solution for 1 h. Subsequently, they were rinsed three times more with 0.1 M phosphate buffer solution. Sequential dehydration was performed using ethanol solutions of 50%, 70%, 90%, and 100% (*v*/*v*) concentrations, with each step lasting for 15 min. Following dehydration, the specimens underwent embedding and curing procedures. Ultrathin sections were then prepared using an ultramicrotome (Ultracut E, Reichert-Jung, Vienna, Austria), and stained with 2% (*w*/*v*) uranyl acetate and 0.08 M lead citrate. Finally, algal cells were observed by transmission electron microscope (JEM-1200, JEOL, Tokyo, Japan), with photographic documentation [17].

### 4.6. Measurement of Chlorophyll Fluorescence

Chlorophyll fluorescence parameters (Fv/Fm, Y(II), and NPQ) were measured using a modulated chlorophyll fluorometer Dual-PAM-100 (Heinz Walz GmbH, Effeltrich, Germany) equipped with a DR detector. Prior to measurement, algal samples were dark-adapted for 10 min [60,61]. The measuring light intensity was set to 24 µmol photons m^−2^ s^−1^ for *U. prolifera* and 40 µmol photons m^−2^ s^−1^ for *S. horneri*. Actinic red light (625 nm) was applied at 127 µmol photons m^−2^ s^−1^ during the measurements. All measurements were performed at 25 °C under consistent experimental settings.

### 4.7. Measurement of Enzyme Activities

Enzyme activities of carbonic anhydrase (CA, product number: CAS-1-W), ribulose-1,5-bisphosphate carboxylase (Rubisco, product number: RUBPS-1-Y) and nitrate reductase (NR, product number: NR-1-W) were measured using assay kits (Suzhou Keming Biotechnology Co., Ltd., Suzhou, China). Freshly harvested algal samples (0.1 g) were homogenized with 1 mL of extraction solution using a grinding machine (JXFSTPRP-CL, Shanghai Jingxin Industrial Development Co., Ltd., Shanghai, China). The homogenate was centrifuged at 8000× *g* and 4 °C for 10 min (TGL-16M, Hunan Xiangyi Laboratory Instrument Development Co., Ltd., Changsha, China), and the supernatant was used for enzyme activity assessment. Enzyme activities of CA, Rubisco and NR were determined using a microplate reader (SynergyMx, BioTek Instruments, Inc., Potton, UK). Absorbance was measured at 405 nm for CA and at 340 nm for Rubisco and NR.

### 4.8. Metabolite Extraction and LC-MS Metabolite Profiling

A total of 100 mg of fresh weight samples of *U. prolifera* and *S. horneri* were ground in EP tubes with liquid nitrogen, respectively. Then, 500 μL of 80% (*v*/*v*) methanol–water solution was meticulously added to each tube. After vortexing for 30 s, the tubes were incubated on ice for 5 min, followed by centrifugation at 15,000× *g* and 4 °C for 20 min. A specific volume of the supernatant was then taken and diluted with mass spectrometry-grade water to attain a methanol content of 53% (*v*/*v*). After that, the diluted samples underwent a further round of centrifugation under identical conditions. The supernatant was then meticulously collected and preserved for LC-MS analysis [62].

The extracts underwent untargeted metabolome analysis using a UHPLC system coupled with a mass spectrometer. The extracts were injected into a Hypesil Gold column (C18) with a column temperature of 40 °C and a flow rate of 0.2 mL min^−1^. In the positive polarity mode, the mobile phase A consisted of 0.1% (*v*/*v*) formic acid, while mobile phase B was methanol. In negative polarity mode mobile phase A comprised 5 mM ammonium acetate adjusted to pH 9.0, and mobile phase B was methanol. The mass spectrometry scan range was set to *m*/*z* 100–1500, utilizing an electrospray ionization (ESI) source with a spray voltage of 3.5 kV. The sheath gas flow rate was maintained at 35 psi, and the auxiliary gas flow rate was set at 10 L min^−1^. The ion transfer tube temperature was set to 320 °C, with an ionization inlet radiofrequency (RF) level of 60. Additionally, the auxiliary gas heater temperature was set to 350 °C. Both positive and negative ion scan modes were selected for polarity, with MS/MS second-level scans conducted in a data-dependent scanning.

### 4.9. Data Analysis

Statistical analysis of the data was performed using SPSS statistical software (version 26.0). Due to the data not meeting the assumptions of homogeneity of variance and normal distribution, the significance of the differences in RGR, chlorophyll fluorescence parameters, and enzyme activities between the experimental and control groups were analyzed using a Student’s *t*-test. GraphPad Prism 8 were utilized for visualization.

Raw metabolomic data, obtained through UHPLC-MS/MS analyses, were processed using Compound Discover 3.1 software. Initial screening of metabolites was conducted based on retention time and mass-to-charge ratio. Peak alignment, extraction, and quantification of peak areas were performed for each sample. Molecular formulas were predicted using molecular ion peaks and fragment ion peaks. Comparative analysis against databases such as Masslist, mzCloud, and mzVault was conducted. Following standardization processing, relevant information regarding metabolites was obtained.

Metabolites were annotated using KEGG, HMDB, and LIPID Maps databases. Data transformation was performed using meta X software (version 1.4.16) [63]. Multivariate statistical analysis was conducted using principal component analysis (PCA) and partial least squares discriminant analysis (PLS-DA) to obtain the variable importance in projection (VIP) scores for the metabolites. Univariate analysis was performed based on Student’s *t*-test to determine inter-group statistical significance (*p*-values) and calculate fold changes (FC) between groups. Differential metabolites (DMs) were selected based on VIP values, *p*-values, and FC values. The threshold levels for screening DMs between the experimental and control groups were set at VIP > 1.0, and *p* < 0.05 [64,65,66]. DMs were clustered, and relevant metabolic pathways were analyzed using KEGG database. Data analysis and visualization were completed using Python 3.5.0 and R 3.4.3.

## 5. Conclusions

This study explored the allelopathy between *U. prolifera* and *S. horneri* and their metabolomic responses, revealing their mutual effects on RGR, cell ultrastructure, chlorophyll fluorescence parameters, enzyme activities, and DMs. The results indicated that allelopathy existed between *U. prolifera* and *S. horneri*, but the effects varied. *S. horneri* filtrate significantly inhibited RGR of *U. prolifera*, while *U. prolifera* filtrate had no significant effect on RGR of *S. horneri*. Both algal filtrates caused varying degrees of damage to the ultrastructure of the other alga. The photosynthesis system was a major target of allelopathy, with significant impacts on the chlorophyll fluorescence parameters, CA, and Rubisco activity. Algal filtrates also significantly promoted the activity of NR. Additionally, differential metabolite analysis and KEGG topological analysis revealed significant changes in metabolites related to amino acid, nucleotide metabolism, and pentose phosphate pathways. Based on the findings above, it was hypothesized that *U. prolifera* and *S. horneri* might have different mechanisms for responding to allelopathy. *U. prolifera* likely relied mainly on its internal compensation mechanisms to cope with allelopathic pressure, while *S. horneri* may primarily depend on its own defense strategies. These findings not only enhance our understanding of the allelopathic mechanisms between green and golden tides but also provide new theoretical foundations for the control of HABs.

## Data Availability

Data will be made available on request.

## Supplementary Materials

The following supporting information can be downloaded at: https://www.mdpi.com/article/10.3390/plants13212966/s1, Figure S1: Principal component analysis (PCA) of metabolites in *U. prolifera* (A) and *S. horneri* (C) groups; Partial least squares-discriminate analysis (PLS-DA) of metabolites in *U. prolifera* (B) and *S. horneri* (D) groups; Figure S2: Volcano maps of intergroup differential metabolites in *U. prolifera* (A) and *S. horneri* (B) groups; Figure S3: Algal samples used in the experiments collected in June 2022. *U. prolifera* samples from Xiaomai Island and Lingshan Island, Qingdao (A,C); *S. horneri* samples from the kelp aquaculture area in Li Island, Rongcheng (B,D). C shows reproductive cells in *U. prolifera* tissues, while D shows the receptacles of *S. horneri*. Table S1: List of DMs in *U. prolifera* identified by UHPLC; Table S2: List of DMs in *S. horneri* identified by UHPLC.
