# Supplementary material for: Allelopathic Interactions Between the Green-Tide-Forming Ulva prolifera and the Golden-Tide-Forming Sargassum horneri Under Controlled Laboratory Conditions
Source: Plants (Basel). 2024 Oct 24;13(21):2966. doi: 10.3390/plants13212966 (PMC11548249; doi:10.3390/plants13212966)
Supplement: Supplementary file 1 [file plants-13-02966-s001.zip › Figures.pdf]

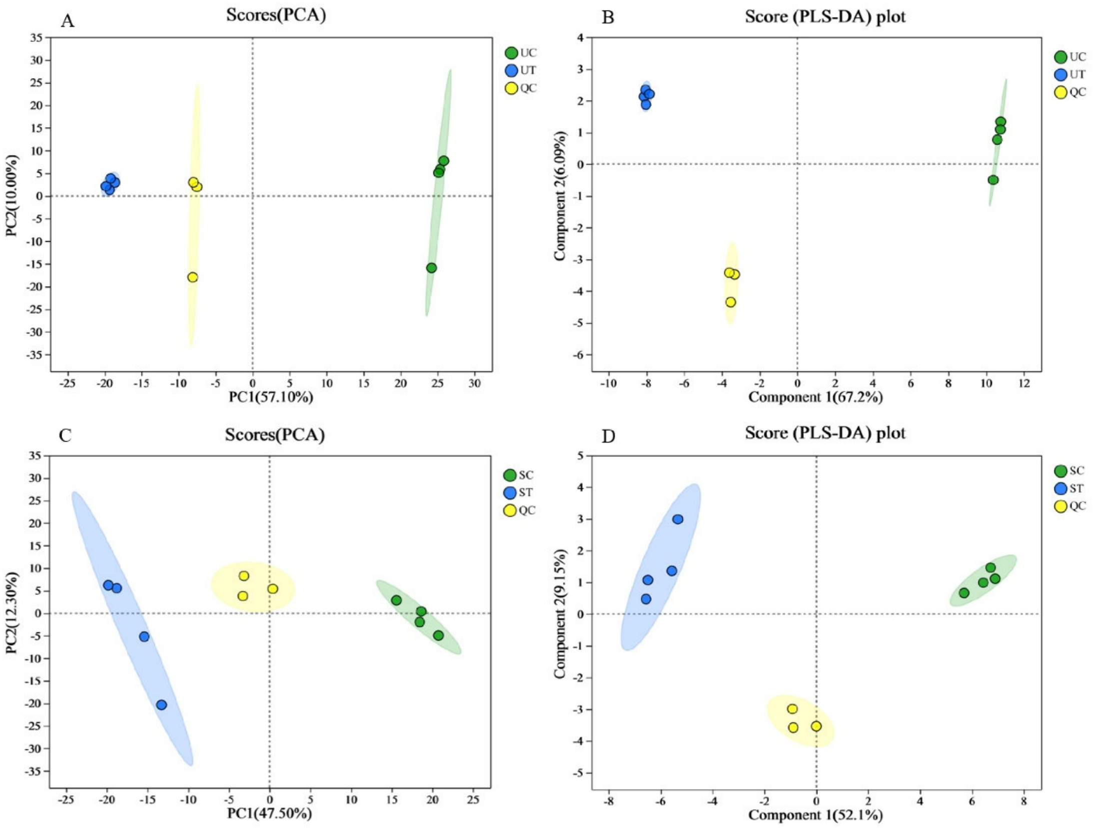

Figure S1 Principal component analysis (PCA) of metabolites in *U. prolifera* (A) and *S. horneri* (C) groups; Partial least squares-discriminate analysis (PLS-DA) of metabolites in *U. prolifera* (B) and *S. horneri* (D) groups.

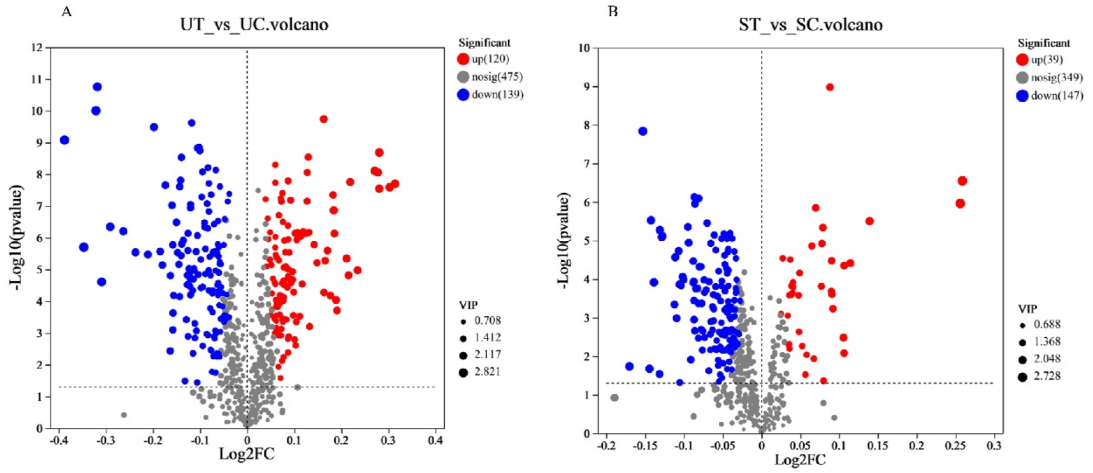

Figure S2 Volcano maps of intergroup differential metabolites in *U. prolifera* (A) and *S. horneri* (B) groups.

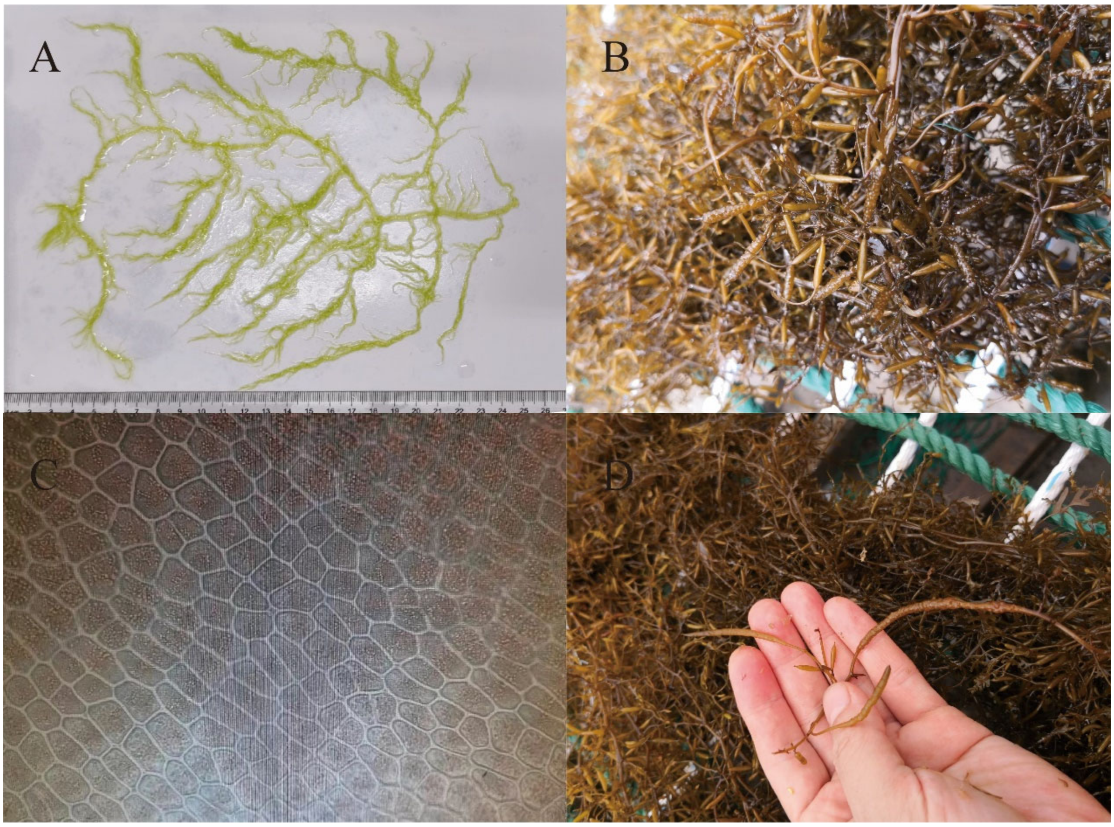

Figure S3 Algal samples used in the experiments collected in June 2022. *U. prolifera* samples from Xiaomai Island and Lingshan Island, Qingdao (A, C); *S. horneri* samples from the kelp aquaculture area in Li Island, Rongcheng (B, D). C shows reproductive cells in *U. prolifera* tissues, while D shows the receptacles of *S. horneri*.
