# Supplementary material for: Allelopathic Interactions Between the Green-Tide-Forming Ulva prolifera and the Golden-Tide-Forming Sargassum horneri Under Controlled Laboratory Conditions
Source: Plants (Basel). 2024 Oct 24;13(21):2966. doi: 10.3390/plants13212966 (PMC11548249; doi:10.3390/plants13212966)
Supplement: Supplementary file 1 [file plants-13-02966-s001.zip › Tables.pdf]

Table S1 List of DMs in *U. prolifera* identified by UHPLC.

Metabolite	Up.Down	VIP	FC	Pvalue
4-(2,3-dihydro-1,4-benzodioxin-6-yl)butanoic acid	down	2.8211	0.7864	1.98E-06
Monobutyl phthalate	down	2.6963	0.8005	9.98E-11
methyl 2-[(2-acetyl-3-oxo-1-butenyl)amino]acetate	down	2.6688	0.7644	8.37E-10
Pyridoxine	down	2.5	0.8022	1.77E-11
Methyl arachidonate	down	2.4854	0.8073	2.46E-05
all-cis-4,7,10,13,16-Docosapentaenoic acid	up	2.3679	1.2148	2.08E-09
Ruscogenin	up	2.3327	1.2431	2.00E-08
vasicine	up	2.2902	1.2334	2.58E-08
5'-Deoxy-5'-(Methylthio)Adenosine	down	2.2677	0.8176	4.53E-07
Theobromine	up	2.2137	1.2148	2.85E-08
4-Hydroxymephenytoin	up	2.1756	1.2121	8.74E-09
3-Indoleacetonitrile	up	2.1705	1.2065	7.91E-09
DL-Arginine	up	2.117	1.1359	1.37E-07
4-hydroxy-1-methyl-3-(phenylthio)-1,2-dihydroquinolin-2-one	down	2.0917	0.8334	6.23E-07
Glu-Gln	up	2.0778	1.1577	4.49E-06
PC (14:1e/4:0)	down	2.067	0.8719	3.29E-10
(+/-)10(11)-EpDPA	down	2.0565	0.8485	2.88E-06
UDP	up	2.0344	1.1766	1.06E-05
N6-Succinyl Adenosine	up	2.0178	1.1638	1.76E-08
Icariin	down	1.9698	0.8641	3.39E-06
Sinapyl Alcohol	up	1.9636	1.1611	1.53E-05
Proscillaridin A	up	1.9316	1.1398	9.11E-05
D-Gluconic acid	up	1.931	1.1194	1.83E-10
LPC 18:1	down	1.8979	0.8799	2.71E-06

DGDG (16:4/18:4)	up	1.8789	1.137	7.40E-07
PC (16:2e/2:0)	down	1.8758	0.8953	9.53E-08
LDGTS 19:1	down	1.8712	0.8866	2.22E-08
Uridine diphosphate glucose	up	1.8452	1.1256	2.54E-06
Methyl linoleate	up	1.8267	1.1414	0.000196
Vincamine	down	1.8225	0.8828	7.24E-06
PC (18:4e/2:0)	down	1.8022	0.9012	3.30E-07
Galloylpaeoniflorin	up	1.8018	1.1304	6.61E-05
1-ethyl 4-(2-oxo-1,2-diphenylethyl) succinate	up	1.789	1.1217	5.29E-06
LSD-d3	down	1.7802	0.8968	0.000235
LPC(1-acyl 16:0)	down	1.7796	0.9056	2.47E-08
PC (14:1e/2:0)	down	1.7725	0.9078	2.95E-09
LPE 16:1	down	1.7335	0.9082	1.58E-06
N-Palmitoyl taurine	up	1.7326	1.1198	5.28E-05
LysoPC 20:4	down	1.7242	0.893	0.003681
LPC 22:6	down	1.7232	0.9068	1.56E-08
FAHFA (18:2/20:4)	down	1.7182	0.8965	1.68E-06
Salvianolic acid D	up	1.7156	1.1349	4.49E-08
Coenzyme Q2	up	1.7029	1.0902	7.32E-07
Lenalidomide	down	1.7024	0.92	1.15E-07
Hordatine A	up	1.6921	1.0942	2.90E-09
LPE 15:1	down	1.6854	0.9028	2.93E-06
14,15-Dehydrocrepenynic acid	down	1.685	0.9217	2.41E-10
Lysopc 14:0	down	1.6811	0.8965	0.0008
ethyl 4-[(5-chloro-3-pyridyl)oxy]-5,8-difluoroquinoline-3-carboxylate	down	1.6746	0.8933	1.57E-05
10-Gingerol	up	1.6667	1.0767	6.84E-07

LPE 15:0	down	1.6586	0.9044	6.93E-06
1,2-dihydroxyheptadec-16-yn-4-yl acetate	up	1.6534	1.0817	9.01E-07
LDGTS 18:4	down	1.6488	0.9328	1.82E-09
L-Ornithine	up	1.648	1.1036	1.65E-06
(+/-)13-HODE	down	1.6376	0.9293	1.50E-09
LDGTS 18:0	down	1.6323	0.9055	7.14E-05
Nicotinate ribonucleoside	down	1.6297	0.8975	6.55E-05
LPC 18:2	down	1.6293	0.9103	1.22E-06
L(-)-Carnitine	down	1.6262	0.9198	1.99E-05
Octadecanedioic acid	down	1.6199	0.9318	1.41E-09
2-piperidinobenzoic acid	down	1.6186	0.9166	7.31E-07
LPC(1-acyl 18:3)	down	1.6001	0.9201	1.14E-07
Triethyl citrate	down	1.5977	0.9075	3.66E-06
PC (18:5e/2:0)	down	1.5967	0.92	8.97E-08
IAA-Asp	up	1.5962	1.1083	6.14E-06
2-hydroxy-6-[(8Z,11Z)-pentadeca-8,11,14-trien-1-yl]benzoic acid	down	1.5961	0.9277	2.73E-06
Glycerophospho-N-palmitoyl ethanolamine	down	1.5894	0.9164	2.53E-06
Arachidonic acid	up	1.585	1.0964	0.000628
Carbaprostacyclin	up	1.5706	1.0622	1.63E-08
3-Dehydroshikimic acid	up	1.5692	1.086	6.43E-07
L(-)-Malic acid	up	1.5642	1.0952	6.77E-07
1-(3-ethyl-2,4-dihydroxy-6-methoxyphenyl)butan-1-one	up	1.5642	1.0852	7.82E-07
3-Hydroxy-glabrol	down	1.5629	0.9144	0.000381
LysoPE 18:2 (2n isomer)	down	1.5614	0.9157	1.52E-05
Citric acid	up	1.5457	1.0662	6.60E-08
Pheophorbide A	down	1.536	0.9365	8.40E-09

Euphorbia factor L1	down	1.5355	0.9228	4.59E-05
Lysopc 20:0	down	1.5249	0.9181	9.36E-06
Arachidic Acid	down	1.5242	0.9156	3.01E-05
L-Citrulline	up	1.5183	1.0844	2.88E-05
N2,N2-Dimethylguanosine	up	1.5073	1.0923	7.09E-08
Lysope 16:0	down	1.5035	0.92	6.34E-05
LLK	down	1.4999	0.9189	1.51E-05
Epitestosterone	up	1.4994	1.069	1.17E-05
Ala-Val	up	1.4911	1.0925	8.81E-09
LPC 16:1	down	1.481	0.9248	9.42E-06
Gamma-Glu-Leu	down	1.4796	0.9305	1.44E-05
4-Guanidinobutyric acid	up	1.4755	1.0711	5.17E-05
17(S)-HpDHA	down	1.4721	0.9281	0.000857
Indole-3-acetic acid	down	1.4687	0.9192	3.35E-05
1,7-bis(3,4-dihydroxyphenyl)heptan-3-one	up	1.465	1.0752	0.000447
4-Aminobutyric acid	up	1.4626	1.0731	7.26E-07
5(S),15(S)-DiHETE	up	1.4616	1.0692	2.09E-05
LPE 20:4	down	1.4606	0.9168	0.001278
(+)-Tetrandrine	up	1.4588	1.0806	0.000298
Lysope 14:0	down	1.4479	0.9241	2.00E-05
Lysopg 18:1	down	1.4478	0.9127	0.03255
6-Methylquinoline	down	1.4406	0.9354	5.22E-08
L-Asparagine	up	1.4322	1.0703	0.00028
3-Furoic acid	up	1.4304	1.0759	1.17E-06
(3beta,9xi)-3-(beta-D-Glucopyranosyloxy)-14-hydroxycard-20(22)-enolide	down	1.4299	0.9367	3.57E-05
Trigonelline	down	1.4289	0.945	1.08E-05

FAHFA (18:1/20:3)	down	1.4248	0.9294	8.56E-07
LDGTS 22:4	down	1.4213	0.9441	6.17E-09
Corticosterone	down	1.4157	0.9397	4.66E-07
Testosterone	up	1.4137	1.0553	2.27E-05
Hordatine B	up	1.4107	1.0611	3.67E-05
Indole	down	1.41	0.9392	3.36E-07
Thromboxane B2	up	1.4039	1.0562	3.07E-05
LDGTS 17:2	down	1.3924	0.9428	8.41E-08
4-Guanidinobutanoic acid	up	1.392	1.0664	2.69E-05
5-Hydroxyindole-3-acetic acid	down	1.3902	0.9254	1.08E-05
Licochalcone A	down	1.3864	0.9405	2.26E-06
Senecionine	up	1.3855	1.0622	9.87E-06
Prostaglandin K2	down	1.3811	0.951	2.84E-06
Indole-3-acrylic acid	down	1.3787	0.9485	4.40E-08
5-[(8Z,11Z)-pentadeca-8,11-dien-1-yl]benzene-1,3-diol	up	1.3739	1.0724	0.001615
LPE 14:0	down	1.3739	0.9235	0.004385
Scopolamine	down	1.37	0.9259	0.000501
16-Hydroxyhexadecanoic acid	up	1.3674	1.0584	8.01E-06
Dihydrothymine	up	1.3673	1.0636	1.88E-05
beta-Nicotyrine	down	1.3672	0.9343	3.80E-05
LPE 20:5	down	1.3647	0.9261	0.001432
Tigloylgomisin H	down	1.3612	0.9342	4.84E-06
Medroxyprogesterone	down	1.3535	0.9453	1.49E-07
Hirsutine	up	1.339	1.0632	0.001298
octadec-9-ynoic acid	down	1.3329	0.9509	4.65E-05
Vitamin B2	up	1.3323	1.0641	2.90E-06

Ophiopogonin D	up	1.3308	1.0564	1.23E-06
L-Glutamic acid	up	1.3301	1.0514	0.000747
L-Cysteine-glutathione gisulfide	up	1.3269	1.0579	2.86E-06
Guan-fu base A	up	1.3267	1.0619	4.13E-07
12-epi Leukotriene B4	down	1.3213	0.9437	5.07E-07
Sclareolide	down	1.3192	0.9453	1.94E-08
Gallic acid	down	1.3176	0.9291	0.03636
(+/-)19(20)-DiHDPA	up	1.3007	1.0519	7.28E-08
15(S)-HpEPE	up	1.2892	1.048	1.96E-07
1-Methylguanine	up	1.2891	1.0619	1.70E-05
L-Cystine	down	1.2805	0.9403	1.38E-05
LDGTS 16:2	down	1.2774	0.955	7.41E-09
LNK	up	1.277	1.0622	0.004133
Fraxinellone	down	1.2758	0.9367	0.002564
Miltirone	up	1.2741	1.0742	0.002469
LysoPC 15:0	down	1.2735	0.9452	4.56E-05
Prostaglandin J2	down	1.2731	0.9526	3.73E-07
Spectinomycin	down	1.27	0.9593	1.26E-05
D-Raffinose	down	1.2664	0.9554	3.10E-06
LPC 15:0	down	1.2635	0.9472	0.000167
L-Threonic acid	up	1.2614	1.0444	4.16E-06
Norfludiazepam	up	1.2566	1.044	0.000147
Alisol B 23-acetate	down	1.2535	0.9423	0.00108
YLH	up	1.2534	1.0541	3.18E-07
Sulforidazine	up	1.2452	1.0548	9.69E-05
4-Hydroxy-2,5-dimethyl-3(2H)furanone	up	1.2448	1.0534	3.95E-08

LDGTS 22:5	down	1.2437	0.9608	2.44E-08
Deoxyguanosine	up	1.2401	1.0624	1.13E-06
Solasonine	up	1.2369	1.0526	6.95E-05
Tanespimycin	up	1.2336	1.0527	2.70E-05
Phylloquinone	up	1.2327	1.0593	0.000379
15-OxoEDE	up	1.2316	1.0424	5.07E-09
4-Methylcatechol	down	1.2302	0.9525	3.24E-06
(+/-)11-HETE	up	1.2274	1.0424	1.85E-08
Hydroxycitric acid	up	1.2266	1.0499	0.000114
16,16-Dimethyl prostaglandin A1	down	1.2265	0.9505	0.000222
Gingerglycolipid B	up	1.2261	1.0554	7.53E-05
12,13-EODE	down	1.2238	0.95	5.63E-05
N1-{3-[2-(2-pyridyl)ethyl]phenyl}-4-chlorobenzene-1-sulfonamide	up	1.2194	1.062	0.001528
2-Methylpentanedioic acid	up	1.2184	1.0508	5.83E-08
LysoPC 18:0	down	1.2183	0.9477	1.55E-05
L-Tryptophan	down	1.2152	0.9463	3.97E-06
Guanine	up	1.2148	1.0465	0.000148
DGDG (18:3/16:4)	up	1.2117	1.0478	5.14E-06
Lathyrol	down	1.2093	0.9486	0.003576
3-Indolepropionic acid	down	1.2089	0.9439	0.001172
Asp-glu	down	1.2085	0.957	3.09E-06
15-keto Prostaglandin E1	down	1.2022	0.9583	2.96E-07
Embelin	up	1.1985	1.0434	9.08E-05
5-fluoro AB-PINACA N-(4-hydroxypentyl) metabolite	down	1.1966	0.9622	3.56E-05
N1-(2,6-dimorpholino-3-pyridyl)-4-methylbenzamide	down	1.1934	0.9562	0.005539
Glycyl-L-leucine	up	1.1895	1.0554	8.66E-06

Dihydrokawain	up	1.1889	1.053	0.000352
Tectorigenin	up	1.1835	1.0423	2.86E-05
3-Methyl-2-Oxobutanoic Acid	up	1.182	1.0544	2.67E-05
KPH	up	1.1815	1.0341	4.84E-06
MGDG (18:3/16:4)	up	1.1796	1.0431	3.50E-06
Saikosaponin B4	up	1.1783	1.0449	0.000116
Docosahexanoic acid	down	1.176	0.9588	3.88E-06
Choline	down	1.1735	0.9583	2.64E-05
Atractylenolide I	down	1.1726	0.9549	0.00086
Yamogenin	down	1.1719	0.9536	2.68E-06
LysoPC 15:1	down	1.1706	0.9548	1.10E-05
Linoleoyl Ethanolamide	down	1.1699	0.9514	0.005432
Polygalic acid	up	1.1685	1.0541	0.00044
Adenosine 5'-monophosphate	down	1.1664	0.9578	1.62E-06
8-Hydroxyquinoline	down	1.1592	0.9571	3.07E-05
OxPG (18:1-20:5+3O(2Cyc))	up	1.1589	1.0493	0.000265
INK	up	1.1558	1.0552	0.005725
VLH	up	1.1552	1.0441	0.000281
Pyrogallol	up	1.153	1.0478	0.001196
3-hydroxy-3-methylpentanedioic acid	down	1.1522	0.959	0.004616
Maltotetraose	down	1.149	0.959	4.79E-05
Deacetyltaxol	up	1.1488	1.0475	9.96E-05
Ginsenoside F1	up	1.1487	1.0425	9.39E-07
Azetidine-2-carboxylic acid	up	1.1475	1.0527	0.007545
3-Hydroxyhippuric Acid	down	1.1403	0.9478	0.003357
Nobiletin	up	1.1385	1.0494	6.39E-05

Methyl oleate	down	1.1193	0.9533	0.001483
(5E)-7-methylidene-10-oxo-4-(propan-2-yl)undec-5-enoic acid	down	1.1188	0.962	6.84E-06
LDGTS 20:5	down	1.1155	0.9676	2.59E-07
Yuheinoside	up	1.1153	1.0387	1.94E-05
Tretinoin	down	1.1104	0.9523	0.001291
(2E,4E)-N-(2-methylpropyl)dodeca-2,4-dienamide	down	1.1023	0.9665	0.000445
Kahweol	down	1.1006	0.9604	0.005573
Lactose	down	1.0993	0.9691	0.000284
1-Palmitoylglycerol	down	1.0977	0.972	7.27E-08
D-(+)-Proline	up	1.0957	1.0294	8.02E-06
D-(+)-Maltose	down	1.0952	0.9692	0.000284
Tangeretin	up	1.0932	1.0513	0.001207
Thymidine	up	1.0844	1.0421	2.03E-06
3-Hydroxy-3-Methylpentane-1,5-Dioic Acid	up	1.0842	1.0423	0.00117
L-Saccharopine	up	1.0836	1.0413	0.000361
Rauwolscine hydrochloride	up	1.0813	1.0414	2.56E-05
L-Aspartic acid	up	1.0813	1.0357	7.01E-06
7-O-Ethylmorroniside	up	1.0799	1.0463	0.001076
Ergocalciferol	down	1.0794	0.9618	0.00036
Betulonic acid	down	1.0767	0.9558	0.001003
13,14-Dihydro prostaglandin E1	down	1.0742	0.9662	1.69E-06
Oroxin B	down	1.0736	0.9539	0.000851
LPC 20:5	down	1.0685	0.9581	6.49E-05
2,3-dihydroxypropyl 12-methyltridecanoate	up	1.0683	1.0372	0.000185
N-p-Coumaroylspermidine	down	1.0659	0.9614	0.000914
Momordin Ic	up	1.0656	1.0441	0.000801

Praeruptorin B	down	1.0638	0.9607	5.74E-06
DGDG (18:2/16:3)	up	1.0572	1.0505	0.02634
3-Hydroxybutyric acid	down	1.057	0.9602	6.72E-06
N2-Methylguanosine	up	1.0565	1.0417	0.000356
Poricoic acid A	down	1.0543	0.9597	0.000133
Swainsonine	down	1.05	0.9564	0.004416
Methyl myristate	down	1.0491	0.9592	2.00E-06
ethyl 3-[3,5-di(trifluoromethyl)anilino]-2-nitroacrylate	up	1.0452	1.0367	7.28E-06
16,16-Dimethyl prostaglandin A2	down	1.0444	0.9648	5.55E-05
(+/-)12(13)-DiHOME	up	1.0441	1.0275	6.10E-08
DGDG (16:3/18:3)	up	1.0411	1.0344	6.78E-07
Kirenol	up	1.0394	1.032	1.11E-06
Arecoline	down	1.0359	0.9496	0.01674
3,14-dihydro-15-keto-tetranor Prostaglandin E2	down	1.0357	0.9688	0.000371
(20R)Ginsenoside Rg3	up	1.0303	1.0439	0.000415
2-Thio-acetyl MAGE	down	1.0291	0.9668	0.000273
Roseoside	up	1.028	1.0308	2.48E-07
Protobioside	down	1.0256	0.955	0.01761
2'-O-Methylguanosine	up	1.0252	1.0439	0.000685
LDGTS 18:2	down	1.0178	0.9735	0.000315
Artemether	up	1.0118	1.0473	0.01163
alpha-Hederin	down	1.0066	0.9646	0.000147
Cytidine	up	1.0061	1.0374	5.69E-05
alpha-Ketoglutaric acid	down	1.0053	0.9703	6.11E-05
PLH	up	1.005	1.0349	0.000273
Desthiobiotin	up	1.0046	1.0416	0.001

Elaidic acid	down	1.0038	0.974	4.15E-08

Table S2 List of DMs in *S. horneri* identified by UHPLC.

Metabolite	Up.Down	VIP	FC	Pvalue
N1-isopropyl-2-(phenylthio)benzamide	up	2.7281	1.1942	1.09E-06
Dl-Dihydrosphingosine	up	2.7041	1.1965	2.83E-07
Monobutyl phthalate	down	2.4021	0.8994	1.46E-08
Tricin 5-O-hexoside derivative	down	2.1527	0.9084	0.0001213
PS (17:0/17:1)	down	2.1289	0.8887	0.01836
Cinchophen	up	2.0795	1.1014	3.14E-06
4-(2,3-dihydro-1,4-benzodioxin-6-yl)butanoic acid	down	2.0658	0.9132	5.32E-06
Estriol	down	2.0476	0.906	2.98E-06
2-(4-methylphenyl)imidazo[1,2-a]pyrimidine	down	2.0339	0.9147	8.14E-06
Quercetin 3-alpha-L-arabinofuranoside (Avicularin)	down	2.0065	0.9048	0.02107
16,16-Dimethyl prostaglandin A2	down	1.9692	0.9258	2.71E-05
gamma-Glutamylglutamic acid	down	1.9224	0.9287	1.86E-05
(-)-secoisolariciresinol	down	1.8934	0.9326	0.0001021
Nitenpyram	down	1.8735	0.9154	7.66E-06
Coniine	down	1.8735	0.9319	8.70E-05
Wedelolactone	up	1.8727	1.076	0.003282
gamma-Glutamylmethionine	down	1.8711	0.9413	0.0001152
O-Phosphorylethanolamine	down	1.8496	0.937	1.14E-05
beta-Acetoxyisovalerylalkannin	down	1.8321	0.927	0.001032
L-Phenylalanine	down	1.8319	0.9418	7.46E-07
4-acetyl-4-(ethoxycarbonyl)heptanedioic acid	up	1.827	1.0826	3.85E-05
trans-Cinnamic acid	down	1.8204	0.9423	1.11E-06

DNH	down	1.8191	0.9293	0.0001371
2,6-Diethylaniline	down	1.8133	0.913	0.02884
Indirubin	up	1.8128	1.0766	4.47E-05
PC (18:4e/2:0)	down	1.8013	0.9252	0.0004546
N,N-Dimethylaniline	down	1.7802	0.9453	8.07E-07
3-Methoxy prostaglandin F1alpha	down	1.7628	0.9364	4.43E-06
Arachidonic acid	up	1.7619	1.0564	4.62E-06
ethyl 4-[(5-chloro-3-pyridyl)oxy]-5,8-difluoroquinoline-3-carboxylate	down	1.7374	0.9307	0.0001461
2-Oxoadipic acid	down	1.7319	0.9415	0.001125
Pipecolic acid	down	1.7228	0.948	0.0004271
Adenosine	down	1.7146	0.9388	0.01224
HRH	up	1.7113	1.0649	0.000245
LPG 16:0	up	1.7048	1.0496	1.43E-06
Sattabacin	down	1.6837	0.9415	0.0003591
Scutellarein	up	1.6818	1.0766	0.008269
QLH	down	1.6818	0.9409	0.0001251
Miquelianin	down	1.674	0.9526	3.51E-06
1-(4-methoxyphenyl)propane-1,2-diol	down	1.6738	0.9459	4.77E-05
Quercetin	up	1.6728	1.0659	0.0005843
Bevirimat	up	1.6714	1.0631	1.06E-09
Indole-3-acetic acid	down	1.6682	0.9409	3.42E-05
2-(1,3-benzodioxol-5-yl)-6-chloro-4-phenylquinoline	up	1.6629	1.0554	1.19E-05
Sinapinic Acid	down	1.6444	0.9445	0.0001738
D-Phenylalanine	down	1.6275	0.9452	0.0004294
2-methyl-2,3,4,5-tetrahydro-1,5-benzoxazepin-4-one	up	1.6246	1.0646	3.34E-05
Mangiferin	down	1.6195	0.9421	0.002194

N-Caffeoyl putrescine	down	1.6055	0.943	0.0001775
Norfludiazepam	down	1.5812	0.9501	0.0001045
N-Feruloylagmatine	up	1.5805	1.0644	0.0002091
Griffonilide	down	1.5423	0.9587	7.20E-06
Dehydrocholic acid	down	1.539	0.9557	1.18E-05
Guanine	down	1.5358	0.9543	0.0001168
Palmitoleic Acid	up	1.5148	1.046	1.38E-05
Milbemycin A4 oxime	down	1.5127	0.9475	4.74E-05
2-Amino-1,3,4-octadecanetriol	up	1.4996	1.0549	0.0001533
Polypodine B	down	1.4993	0.9489	0.002221
5-Methoxy-N,N-dimethyltryptamine	down	1.4976	0.9518	0.0001904
3-methyl-4-[2-(2-methylphenyl)hydrazono]-4,5-dihydro-1H-pyrazol-5-one	down	1.4845	0.9475	0.002018
LPC(1-acyl 18:3)	down	1.478	0.9452	0.003393
3-Dehydroshikimic acid	down	1.4706	0.9553	0.002677
1-Methyladenine	down	1.4644	0.958	0.0002515
Desoxycortone	down	1.4442	0.9614	0.0002777
(+)-Dihydrojasmonic acid	down	1.4411	0.9296	0.04802
1-(4-chlorophenyl)-2-phenylethan-1-one	down	1.4351	0.9486	0.006917
Senegenin	down	1.435	0.9504	0.003249
5-(tert-butyl)-2-methyl-N-(5-methyl-3-isoxazolyl)-3-furamide	down	1.4343	0.9572	0.0005132
N'-Formylkynurenine	down	1.4283	0.9599	0.0001564
Theobromine-d6	down	1.4273	0.9504	0.0002221
Kirenol	up	1.405	1.057	0.04302
Kojic acid	down	1.395	0.954	0.02375
Salvianolic acid A	down	1.3873	0.9582	0.0005941
Diflucortolone pivalate	down	1.3867	0.958	0.001199

Ligustilide	down	1.3786	0.9557	0.001268
9-Hydroxy-(10E,12Z,15Z)-Octadecatrienoic Acid	down	1.3714	0.967	6.69E-06
14,15-Dehydrocrepenynic acid	down	1.3682	0.9671	7.17E-06
Abscisic Acid	down	1.3645	0.9625	6.23E-05
Isoeugenol	down	1.3518	0.9572	0.00209
(+/-)-JWH 073 N-(3-hydroxybutyl)-d5	down	1.3485	0.96	1.77E-05
4-Hydroxybenzaldehyde	down	1.3468	0.969	5.17E-05
Methyl jasmonate	down	1.346	0.9649	8.94E-06
Forskolin	down	1.3399	0.9642	0.0003681
2-Isopropylmalic acid	down	1.3387	0.9652	0.0001123
N', N''-DiFeruloylspermidine	down	1.328	0.9591	0.006318
Lysopg 18:1	up	1.3229	1.0346	6.93E-05
4-oxo-5-phenylpentanoic acid	down	1.3224	0.9629	0.002362
Scutellarin	down	1.3184	0.9649	0.0001487
Fucoxanthin	up	1.3166	1.0272	0.0002429
L-Glutathione (reduced)	down	1.3113	0.9677	0.0006262
2,3-Dinor-11beta-prostaglandin F2alpha	down	1.2981	0.9679	0.0002047
Quinic acid	up	1.2951	1.0479	0.01161
Cannabidiolic acid	down	1.294	0.971	5.92E-05
gamma-Glutamate-Cysteine	up	1.2916	1.0337	0.0002637
D-Raffinose	down	1.291	0.9604	0.006814
D-Carnitine	down	1.29	0.9741	8.86E-06
O-Feruloyl 4-hydroxylcoumarin	down	1.2822	0.9676	2.43E-05
Rebaudioside C	down	1.2793	0.962	0.01738
Prostaglandin E2-1-glyceryl ester	down	1.2786	0.9679	0.0001718
LPG 18:1	down	1.2775	0.9699	7.08E-05

Carbadox	down	1.275	0.9696	8.96E-06
(11E,15Z)-9,10,13-trihydroxyoctadeca-11,15-dienoic acid	down	1.2708	0.9678	4.11E-05
DGTS (18:1/20:4)	down	1.2676	0.9657	0.007037
2-(acetyloxy)-3-amino-1-[1,2-di(acetyloxy)ethyl]-3-oxopropyl acetate	down	1.2674	0.9608	0.003931
4-methoxy-6-(prop-2-en-1-yl)-2H-1,3-benzodioxole	up	1.2591	1.04	0.02992
(5Z)-3-aminonon-5-enoic acid	down	1.2429	0.9704	1.96E-05
4-[(4-chlorophenyl)thio]-1-methyl-1H-pyrazolo[3,4-d]pyrimidine	down	1.2386	0.9684	0.00207
Sclareolide	down	1.2381	0.9553	0.006827
(+/-)5(6)-EET Ethanolamide	down	1.2351	0.9702	0.002154
Dihydrotanshinone I	down	1.2341	0.9688	0.0005739
4-(hydroxymethyl)benzoic acid	down	1.234	0.9695	0.0006401
N-Methylleucine	down	1.2329	0.9708	1.67E-05
2-(3,4-dihydroxyphenyl)-3,5,7-trihydroxy-6-methyl-4H-chromen-4-one	down	1.226	0.9695	0.000547
16,16-Dimethyl prostaglandin A1	down	1.2256	0.9723	6.43E-06
Salvianolic acid D	up	1.2227	1.0371	0.005466
4-[2-(2-chloro-6-fluorobenzoyl)hydrazino]benzenesulfonamide	down	1.2224	0.9672	0.002228
N-(9-oxodecyl)acetamide	down	1.2183	0.9707	0.0003784
Ixoside	down	1.2162	0.9638	0.005727
4-Hydroxy-3- methoxyphenylglycol sulfate	down	1.2159	0.9716	0.01394
1-Monopalmitin	down	1.2082	0.9761	0.0003528
5-(6-hydroxy-6-methyloctyl)-2,5-dihydrofuran-2-one	down	1.2062	0.9679	0.002916
Dl-Indole-3-lactic acid	down	1.2036	0.9649	0.00272
Isorhychophylline	down	1.1959	0.9666	0.01524
Muscone	down	1.1924	0.976	1.93E-05
10-Hydroxydecanoic acid	up	1.1883	1.034	0.002329
Rotenone	up	1.1875	1.0411	0.009146

Rebaudioside A	down	1.1863	0.9728	0.001078
1-(3,4-dihydroxyphenyl)-7-(4-hydroxyphenyl)heptan-3-one	down	1.1859	0.9699	0.0007452
LDGTS 16:1	up	1.1857	1.0272	0.0002545
LDGTS 18:2	up	1.1787	1.026	3.12E-05
1H-indole-2,3-dione 3-[N-(5,6-diphenyl-1,2,4-triazin-3-yl)hydrazone]	down	1.1766	0.9648	0.001347
LDGTS 18:1	up	1.1747	1.0286	0.0001206
Furanodiene	down	1.1724	0.962	0.01253
LDGTS 20:3	up	1.1697	1.0287	0.0001624
LDGTS 16:0	up	1.1691	1.027	0.0001506
Artemisinin	down	1.1683	0.9653	0.03618
Diisobutylphthalate	up	1.1677	1.0192	2.89E-05
Isophorone	down	1.1647	0.9774	2.89E-05
Protocatechuic acid	down	1.1634	0.9636	0.01455
Uridine 5'-monophosphate	up	1.1564	1.0281	0.0002289
Protostemonine	down	1.1541	0.9773	0.0002264
Tricin 4'-O-(beta-guaiacylglyceryl) ether	down	1.1529	0.9709	0.000149
L-Argininosuccinate	down	1.1523	0.9637	0.02981
(-)-chimonanthine	down	1.1516	0.9734	0.0005138
Gamma-Glu-Leu	down	1.1477	0.9721	0.003815
Guanidinoethyl sulfonate	down	1.1448	0.9689	0.006654
Glycylproline	down	1.1448	0.9737	0.001976
Guanosine	down	1.1421	0.9715	0.005752
13,14-dihydro-15-keto-tetranor Prostaglandin E2	down	1.1296	0.9715	0.0005225
Adenosine 3'5'-cyclic monophosphate	down	1.1276	0.9682	0.00708
12,13-EODE	down	1.1255	0.9756	0.003517
Palmitoylethanolamide	down	1.111	0.9728	0.0006287

Efetaal	down	1.1005	0.9744	0.0009659
LDGTS 16:2	up	1.0984	1.0249	0.0002608
PE (4:0/16:4)	down	1.097	0.9698	0.02155
Apo-13-zeaxanthinone	down	1.0956	0.9627	0.04514
3-(2-thienyl)-1,2,4-oxadiazole-5-carbohydrazide	down	1.095	0.9723	0.002377
Periplogenin	down	1.0919	0.9702	0.00644
3-hydroxy-3-methylpentanedioic acid	down	1.0898	0.9763	0.003807
D-Gluconic acid	up	1.0897	1.0253	0.006325
Vinpocetine	down	1.0784	0.9778	0.0002146
N2,N2-Dimethylguanosine	down	1.066	0.9773	0.0001714
LPG 18:3	down	1.0655	0.9767	0.0006269
gamma-Glutamylcysteine	down	1.064	0.9736	0.004936
Tanshinone IIA sulfonate	up	1.0607	1.0245	0.005092
6-Phosphogluconic acid	down	1.0604	0.9722	0.007617
Folinic acid	down	1.0547	0.9794	0.002514
3-Indolepropionic acid	down	1.0535	0.9718	0.0009461
Jasmonic acid	down	1.0497	0.976	0.002244
Myricetin 3-O-galactoside	down	1.0476	0.9774	0.0001827
LDGTS 18:4	up	1.0459	1.0237	0.0008869
3-N-Methyl-L-histidine	up	1.0457	1.0251	0.004865
6-Pentyl-2H-pyran-2-one	down	1.0457	0.9753	0.005069
Pogostone	down	1.0453	0.9731	0.007951
octadec-9-ynoic acid	down	1.0421	0.9812	0.0003596
Schisandrin A	down	1.0417	0.9765	0.0009214
3-(2-chlorophenyl)-3-(2,4-difluoroanilino)acrylonitrile	down	1.0399	0.9737	0.02208
4-(pentyloxy)benzene-1-carbohydrazide	down	1.0361	0.9753	0.003264

DL-Stachydrine	down	1.0298	0.9801	0.002702
Tazobactam sodium	down	1.0212	0.9793	0.004799
Coenzyme Q2	down	1.0183	0.9771	0.005409
LPE 16:1	down	1.0178	0.9779	0.0001025
Mosloflavone	down	1.0165	0.9764	0.006009
Oleoyl ethanolamide	down	1.0123	0.978	0.004522
Bicyclo prostaglandin E2	up	1.0065	1.0176	0.000795
5-Acetylsalicylic acid	down	1.0041	0.9776	0.004227
